# 13.2 CANNABIDIOL AS AN ANTIPSYCHOTIC DRUG

**DOI:** 10.1093/schbul/sby014.050

**Published:** 2018-04-01

**Authors:** Francisco Guimaraes, Naielly Rodrigues, Nicole Silva, Felipe Gomes

**Affiliations:** 1 School of Medicine of Ribeirao Preto-University of Sao Paulo; 2 University of Pittsburgh

## Abstract

**Background:**

The phytocannabinoid cannabidiol (CBD) attenuates the psychotomimetic effects produced by high doses of delta-9-tetrahydrocannabinol (THC), the main component of the Cannabis sativa plant. Corroborating this effect, several preclinical and clinical studies indicate that CBD has antipsychotic properties. The mechanisms responsible for these properties, however, remain unknown (Campos et al., Philos Trans R Soc Lond B Biol Sci 367:3364–782013). We have recently found that repeated CBD administration prevents the behavioral impairments, measured in the pre-pulse inhibition, social interaction and novel object recognition tests, induced in mice by repeated treatment (28 days) with the NMDA receptor antagonist MK-801. CBD also prevented the neural (measured by delta-FosB) and microglia activation, and the decrease in the number of parvalbumin-positive neurons, observed in the medial prefrontal cortex (Gomes et al., Int J Neuropsychopharmacol 18(5)2014, Schizophr Res 164:155–63, 2015). Currently, we are investigating if CBD could also reverse these changes once they have been established and the possible mechanisms of this effect.

**Methods:**

Male C57BL/6J mice received intraperitoneal injections of MK-801 (0.25, 0.5 or 1 mg/kg, twice a day) for 7 or 14 days. To determine if these treatments regime would induce acute and long-term deficits, the social interaction (SI) test was performed 1 or 8 days after the end of the MK-801 treatment. Twenty-four hours after the SI, animals were submitted to the novel object recognition (NOR) test. Having established that 14 days of MK-801 induce both acute (24 h after) and long-term (8 days after) behavioral deficits in the SI and NOR tests, we investigated if repeated treatment with CBD (15, 30 or 60 mg/kg daily, i.p.) would reverse these changes. CBD treatment began 24h after the end of the MK-801 treatment and lasted for 7 days. Repeated clozapine (1 mg/kg) was used as a positive control. Forty-eight hours after the last injection, animals were submitted to SI and, 24-h later, to the NOR test. In a second experiment, independent groups of mice received, before each CBD injection, AM251 (a CB1 receptor inverse agonist, 0.1–0.3 mg/kg), AM630 (a CB2 receptor inverse agonist, 0.1–0.3 mg/kg), or the 5HT1A receptor antagonist WAY100635 (0.1–0.3 mg/kg). The data were analyzed by ANOVA followed by the Newman-Keuls test.

**Results:**

MK-801 (0.5 mg/kg) administration for 14 days, but not for 7 days, impaired SI and NOR. Repeated CBD or clozapine treatment reversed these impairments. CB1 and CB2 antagonists (AM251 and AM630, respectively) failed to change CBD effect. However, its effect was blocked by pretreatment with the 5HT1A receptor antagonist WAY100635.

**Discussion:**

Our findings show that a 2-week treatment with the NMDA receptor antagonist MK801 impairs social interaction and novel object recognition, which have been associated with negative and cognitive symptoms of schizophrenia, respectively. These behavioral deficits last for at least one week and are reversed by the atypical antipsychotic clozapine or CBD, reinforcing the proposal that this latter drug has antipsychotic-like properties. CBD effects seem to depend on facilitation of 5HT1A-mediated neurotransmission.

